# Complex II subunit SDHD is critical for cell growth and metabolism, which can be partially restored with a synthetic ubiquinone analog

**DOI:** 10.1186/s12860-021-00370-w

**Published:** 2021-06-12

**Authors:** Aloka B. Bandara, Joshua C. Drake, David A. Brown

**Affiliations:** 1 Department of Human Nutrition, Foods and Exercise, Virginia Tech, Blacksburg, VA 24061-0913 USA; 2 Department of Biomedical Sciences and Pathobiology, Virginia Tech, Blacksburg, VA 24061-0342 USA; 3 Mitochondrial Solutions, LLC, 800 Draper Road, Blacksburg, VA 24060 USA

**Keywords:** Electron transport chain, Complex II, Krebs cycle, Succinate dehydrogenase, SDHD, CRISPR/Cas9, Respiration, Oxygen consumption, Glycolysis, ATP synthesis, Apoptosis, Necrosis, ROS, Idebenone

## Abstract

**Background:**

Succinate dehydrogenase (Complex II) plays a dual role in respiration by catalyzing the oxidation of succinate to fumarate in the mitochondrial Krebs cycle and transferring electrons from succinate to ubiquinone in the mitochondrial electron transport chain (ETC). Mutations in Complex II are associated with a number of pathologies. SDHD, one of the four subunits of Complex II, serves by anchoring the complex to the inner-membrane and transferring electrons from the complex to ubiquinone. Thus, modeling SDHD dysfunction could be a valuable tool for understanding its importance in metabolism and developing novel therapeutics, however no suitable models exist.

**Results:**

Via CRISPR/Cas9, we mutated SDHD in HEK293 cells and investigated the in vitro role of SDHD in metabolism. Compared to the parent HEK293, the knockout mutant HEK293Δ*SDHD* produced significantly less number of cells in culture. The mutant cells predictably had suppressed Complex II-mediated mitochondrial respiration, but also Complex I-mediated respiration. SDHD mutation also adversely affected glycolytic capacity and ATP synthesis. Mutant cells were more apoptotic and susceptible to necrosis. Treatment with the mitochondrial therapeutic idebenone partially improved oxygen consumption and growth of mutant cells.

**Conclusions:**

Overall, our results suggest that SDHD is vital for growth and metabolism of mammalian cells, and that respiratory and growth defects can be partially restored with treatment of a ubiquinone analog. This is the first report to use CRISPR/Cas9 approach to construct a knockout SDHD cell line and evaluate the efficacy of an established mitochondrial therapeutic candidate to improve bioenergetic capacity.

## Background

Mitochondria generate the majority of adenosine triphosphate (ATP) through the electron transport chain (ETC). Succinate dehydrogenase (Complex II; EC 1.3.5.1) uniquely serves as a component of both the Krebs cycle and the ETC [1, 2]. As a component of the Krebs Cycle, Complex II catalyzes oxidation of succinate to fumarate [1, 3], whereas in the ETC, it is one of two entry points for electrons, acquiring electrons from succinate and donating them to ubiquinone (CoQ) [1–3]. Thus, impairments in Complex II function can have severe consequences for maintaining energetic homeostasis. Mutations in Complex II subunits have been found in patients with mitochondrial respiratory deficiency [4], as well as a number of cancers [5–7]. Therefore, an in-depth understanding of Complex II in energetic homeostasis and its viability as a target for treatment is warranted.

Complex II carries four protein subunits [2], all of which are encoded by nuclear genes. Two subunits, SDHA and SDHB, are localized on the matrix side of the inner membrane, and carry the binding site for succinate, three FeS clusters, as well as a flavoprotein bound to an FAD cofactor [8]. The two remaining subunits, SDHC and SDHD, are hydrophobic and anchor the complex to the inner-membrane. Subunits SDHC and SDHD form the CoQ binding site of Complex II and serve as terminal electron transfers from Complex II to CoQ. In particular, mutation of SDHD has been noted in patients with paragangliomas and pheochromocytomas [7]. Expression of truncated SDHD cDNA in Chinese hamster fibroblasts led to increased steady-state levels of superoxide generation, supporting the hypothesis that mutations in SDHD may contribute to carcinogenesis [9]. Therefore, given the particular role of SDHD in anchoring Complex II to the inner-membrane, passaging electrons to CoQ, and noted pathologies, modeling SDHD dysfunction could be a valuable tool for understanding the role of Complex II in metabolism and developing novel therapeutics. To date, however, suitable models for molecular examinations of any Complex II subunit do not exist.

We successfully used a CRISPR/cas9 approach to mutate the SDHD subunit of Complex II in HEK293 cells, and characterized its requirement for mitochondrial respiration, ATP synthesis, and cell growth in vitro. Furthermore, we demonstrate the efficacy of the mitochondrial therapeutic idebenone to improve mitochondrial dysfunction in cells with SDHD mutation. Our results demonstrate the necessity of SDHD for energetic homeostasis and suggests it as a viable target for therapies aimed to improve mitochondrial function.

## Results

### Construction of mutant HEK293Δ*SDHD*

**Fig. 1:**
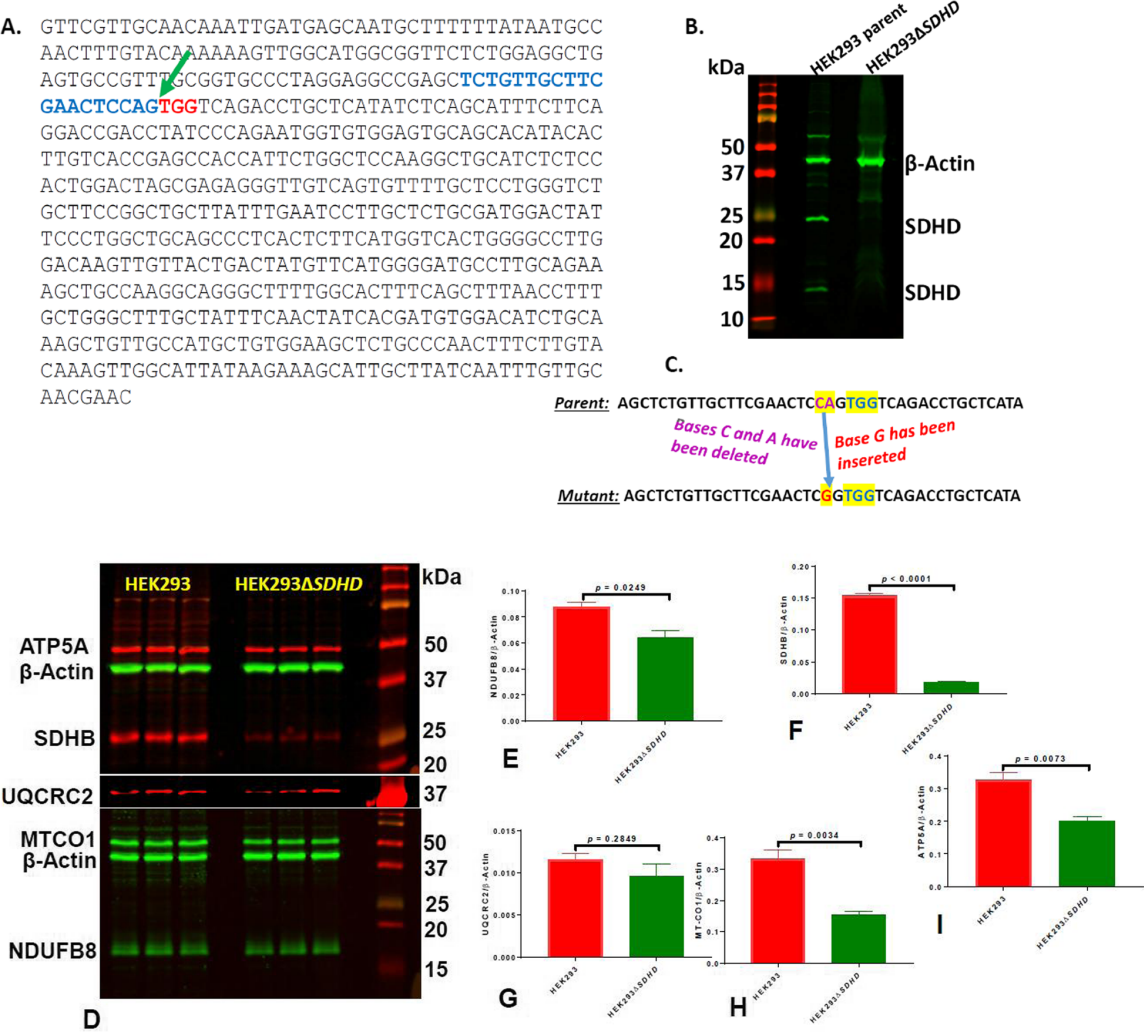
Construction of the SDHD mutant. The gene sequence of SDHD with CRISPR targeting sites is shown **a**. The sgRNA and PAM sequences predicted by the online idtdna program are depicted in blue and red colors, respectively; the specific site for mutation (at 118-bp) is shown with a green arrow. Expression of SDHD protein is shown **b**. The protein extracts reacted with rabbit polyclonal antibodies to SDHD and rabbit polyclonal antibodies to β-Actin are shown. The DNA level recombination events in the genome of the mutant are illustrated **c**. The bases c and a (highlighted and in purplr color) of the parent have been replaced with the base **g** (highlighted and in red color) in the mutant. Expression of representative subunits from all five ETC complexes are shown **d-i**. The expression of NDUFB8 (complex **i**; **d** and **e**), SDHB (comlex II; **d** and **f**), UQCRC2 (complex III; **d** and **g**), MTCO1 (complex IV; **d** and **h**) and ATP5A (complex V; **d** and **i**) were normalized to the expression of β-Actin

The single guide RNA (sgRNA) sequences were designed based on the 609 bp long *SDHD* nucleotide sequence of *Homo sapiens* (GenBank locus ID KR710199.1) using Integrated DNA Technologies’ online tool (Skokie, IL; https://www.idtdna.com/site/order/designtool/index/CRISPR_CUSTOM). A 118-bp site of the forward strand of *SDHD* was predicted as the most reliable for mutation with an on-target specificity score of 68% and off-target score of 58%. The guide strand predicted was TCTGTTGCTTCGAACTCCAG, which is located right upstream of the protospacer adjacent motif (PAM) sequence TGG (Fig. 1a). The constructed mutant HEK293Δ*SDHD* was validated by Western immunoblotting. The predicted molecular weight of SDHD is approximately 17 kDa. Nevertheless, the antibody used for mutant validation was anticipated to produce an additional band of about 29 kDa as well on the blot from parent cells (see the details from the vendor: https://www.lsbio.com/antibodies/cbt1-antibody-sdhd-antibody-n-terminus-wb-western-ls-c345301/356234). Our mutant HEK293Δ*SDHD* was found missing both 17 kDa and 29 kDa protein bands (Fig. 1b).

We then PCR amplified and sequenced the *SDHD* region encompassing the CRISPR targeting site (at 118-bp) of the mutant. The results indicated that an INDEL mutation has been generated at a site two bases upstream of the GTT PAM sequence. Two bases (a C and an A) have been.

deleted from this site and one base (a G) has been inserted at the deletion site (new Fig. 1c). As a result, the whole amino acid sequence downstream of this INDEL site has been altered. Moreover, a stop codon has been inserted 180 bases downstream of the GTT PAM sequence.

Using the online CRISPR prediction site of the Integrated DNA Technologies, we identified the top three potential off-target sites (Table 1). The DNA regions encompassing these sites were PCR amplified and sequenced. The DNA sequences of these sites of the mutant were identical to those of the parent cell line indicating absence of any off-target genetic alterations in the genome of the mutant.

**Table 1:** Targeted mutagenesis site, possible off-target sites, the respective protospacer adjacent motif (PAM) sequences, and forward (F) and reverse (R) oligonucleotide primers used for PCR amplification and DNA sequencing

Guide strand	PAM	Chromosome in human genome	Oligo primers used in sequencing (5′-3′)
Targeted mutagenesis site			
TCTGTTGCTTCGAACTCCAG	TGG	11	GTTCGTTGCAACAAATTGAT (F)
			GTCAGTAACAACTTGTCCAA (R)
Possible off-target sites			
TATG-TGCTTCGAGCTCCAG	AGG	19	CTTCTGTGCCTTACGTACCA (F)
			TGTACTGTAACCAAGTACGC (R)
TCTCTTGCTTC-AACTCCAG	AGG	3	GTAGAGGCAAGGTCTTCCTG (F)
			TTACCAGCCTCCTTGGACCT (R)
TCAGCTTGCTTCGAACTCCTG	GAG	12	TTAGCAATAACTAGTCCAAC (F)
			CAGAGGTCACTGTAGTGTGG (R)

We then investigated whether mutation in *SDHD* altered the expression of other subunits of the five ETC complexes. Expression of complex II subunit SDHB (Fig. 1d and f) decreased significantly in the mutant (*p* < 0.0001) suggesting that SDHD expression has direct impact on expression of other subunits of the complex II. Moreover, expression of complex I subunit NDUFB8 (Fig. 1d and e), complex IV subunit MT-CO1 (Fig. 1d and h), and complex V subunit ATP5A (Fig. 1d and i) decreased significantly in the mutant (*p* = 0.0249, 0.0034, and 0.0073, respectively) suggesting an association in protein expression between complexes II and other ETC complexes. Nevertheless, the expression of complex III subunit UQCRC2 (Fig. 1d and g) was not different between the parent and the mutant suggesting that mutation did not impact expression of this subunit/complex.

### Loss of SDHD increases apoptosis and necrosis

**Fig. 2:**
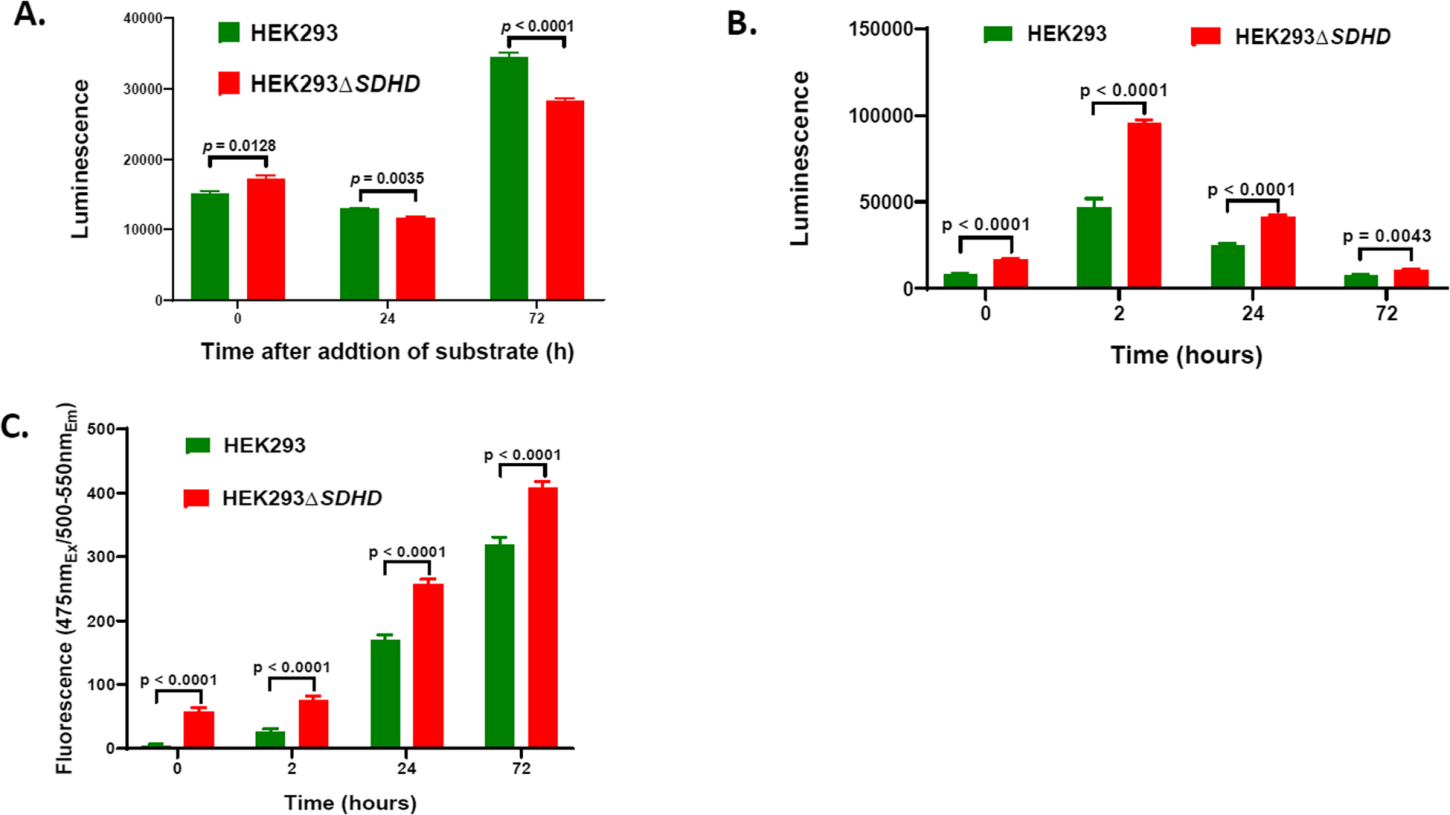
ROS production, apoptosis, and necrosis of cell lines. ROS generation measured using ROS-Glo H_2_O_2_ Assay of parent HEK293 and the mutant HEK293Δ*SDHD* is shown (**A**). The measurements are expressed as total luminescence per 15,000 cells measured at 0, 24, and 72 hours after addition of substrate to the reaction. Apoptosis (**B**) and necrosis (**C**) per 15,000 cells measured using RealTime-Glo Annexin V Apoptosis and Necrosis Assay at 0, 2, 24 and 72 hours of incubation are shown. The apoptosis measurements are expressed as total luminescence, whereas necrosis measurements expressed as total flourescence. Error bars represent the SE of the mean

Compared to the parent HEK293, the mutant HEK293Δ*SDHD* produced significantly increased amount of ROS at 0 h (immediately after addition of the substrate to the reaction; *p* = 0.0128; Fig. 2a). Nevertheless, the mutant produced significantly decreased ROS amounts at 24 h (*p* = 0.0035) and 72 h (*p* < 0.0001) after addition of the substrate. These observations suggest that disruption of *SDHD* results in subsequent decrease of ROS generation over time. It is possible that leakage of electrons due to impaired complex II caused increased ROS production at early stages of cell growth (i.e., 0 h). Nevertheless, the severe impairment of electron transport may have caused decreased overall electron leak and subsequent ROS generation at late stages of growth (i.e., 24 and 48 h). At all the time points, the mutant displayed significantly increased apoptosis (*p* < 0.0001, < 0.0001, < 0.0001, and = 0.0043 respectively at 0, 2, 24, and 72 h post-incubation; Fig. 2b). The mutant also showed significantly increased necrosis (*p* < 0.0001 for each time point; Fig. 2c). Our findings indicate that disruption of *SDHD* made the cells more apoptotic and susceptible to necrosis.

### Loss of SDHD impairs growth

**Fig. 3:**
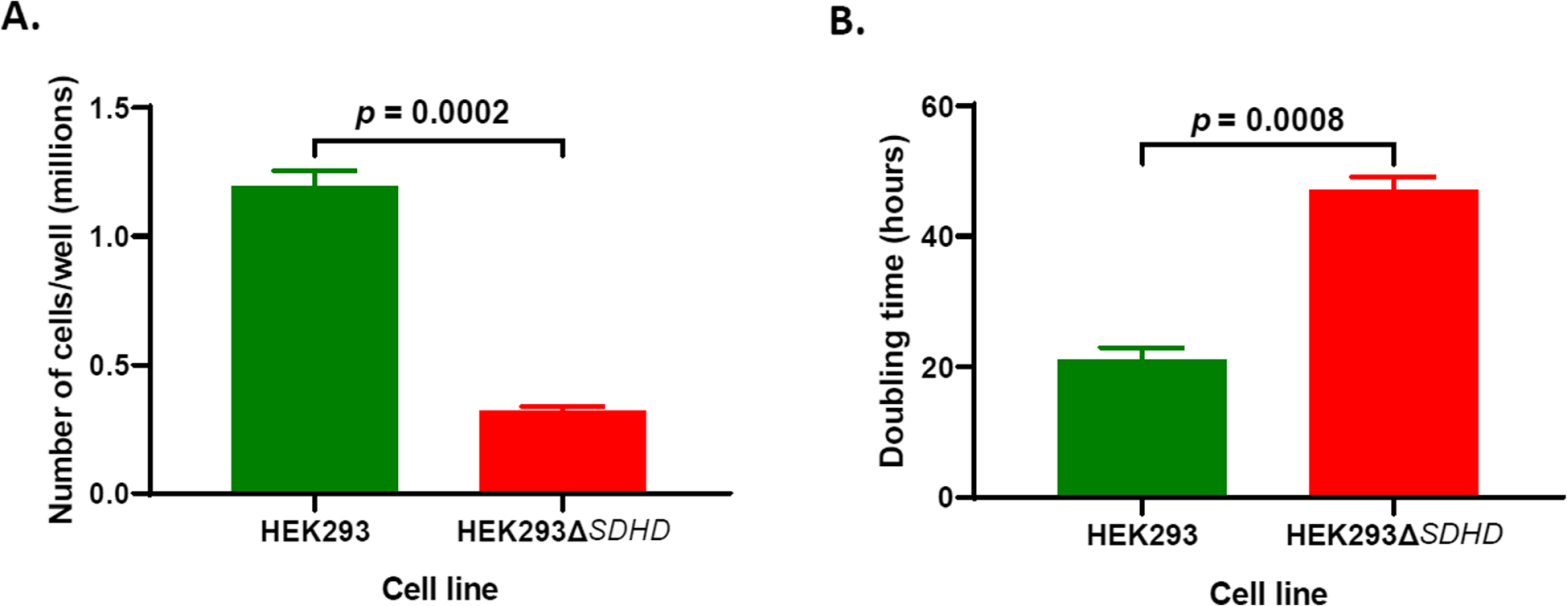
Growth of cell lines. The number of cells harvested from the parent HEK293 and the mutant HEK293Δ*SDHD* cells after four days of the culture (**A**) and doubling times of cells (**B**) are shown. Error bars represent the SE of the man

After 4 days of incubation in growth media, the number of cells recovered from the mutant HEK293Δ*SDHD* culture was ~ 73% less than the amount recovered from parent HEK293 culture (*p* = 0.0002; Fig. 3a). The doubling of mutant over 4 days was significantly slower compared to that of the parent during the same duration of growth (*p* = 0.0008; Fig. 3b), suggesting that SDHD is vital for cell growth.

### Loss of SDHD impairs mitochondrial respiration

**Fig. 4:**
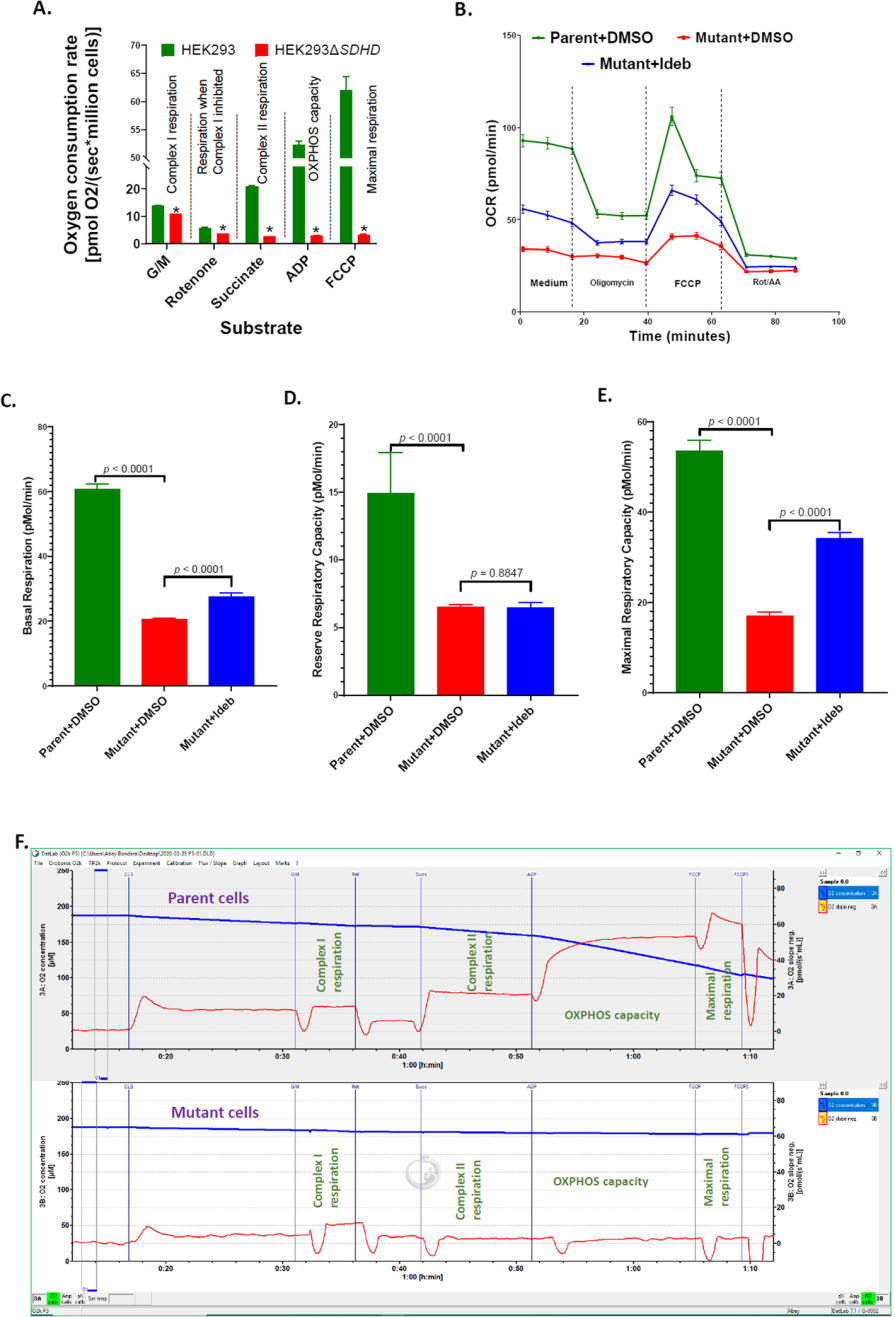
Respiration, of cell lines. Oxygen consumption rates measured using Oroboros O2k per million cells in response to treatment with glutamate-malate (G/M), rotenone, succinate, ADP, and FCCP (**A**) are shown. The mean oxygen consumption rates were compared between the parent HEK293 and the mutant HEK293Δ*SDHD*. The *p* values for the differences between the mean values were 0.0019, 0.0101, 0.0002, 0.0002 and 0.0017, respectively for Complex I respiration, rotenone treatment, Complex II respiration, OXPHOS capacity, and maximal respiration. Oxygen consumption rate (OCR) was measured using Agilent Seahorse XFe96 of parent cells treated DMSO (Parent + DMSO), mutant cells treated DMSO (Mutant + DMSO), and mutant cells treated 1μM idebenone (Mutant + Ideb). OCR per 20,000 permeabilized cells (**B**), and the calculated basal respiration (**C**), reserve respiratory capacity (**D**) and maximal respiratory capacity (**E**) are shown. The mean OCR values were compared between the parent versus the mutant or DMSO treatment versus idebenone treatment. Error bars represent the SE of the mean. Representative Oroboros oxygraphs depicting oxygen consumption of cell lines (**F**) are shown. The top and bottom panels represent parent and mutant cell lines, respectively. The oxygen consumption rate is illustrated by the red line

Oxygen consumption was measured using Oroboros Oxygraph 2 k. Mutation in SDHD decreased oxygen consumption all along the ETC (Fig. 4a and f). Complex I respiration was significantly decreased in mutant cells compared to parent (*p =* 0.0019). Subsequent to rotenone treatment, oxygen consumption of both parent and mutant decreased, but the oxygen consumption of mutant cells was significantly less than that of parent cells (*p =* 0.0101). In response to treatment with succinate, Complex II-mediated oxygen consumption was significantly repressed in mutant cells compared to parent cells *(p* = 0.0002), further confirming the effect SDHD mutation. The mutant also had decreased OXPHOS.

capacity *(p* = 0.0002) and maximal respiration *(p* = 0.0017). Oxygen consumption of permeabilized cells was also assessed using an Agilent Seahorse XFe96 Analyzer (Fig. 4b). In the mutant, basal respiration (*p* < 0.0001; Fig. 4c), reserve respiratory capacity (*p* < 0.0001; Fig. 4d), and maximal respiratory capacity (*p* < 0.0001; Fig. 4e) decreased significantly. Oroboros O2k and Seahorse OCR assays collectively indicate a severe impairment of cellular respiration due to SDHD disruption.

### Loss of SDHD impairs glycolytic capacity and ATP synthesis of cell lines

**Fig. 5:**
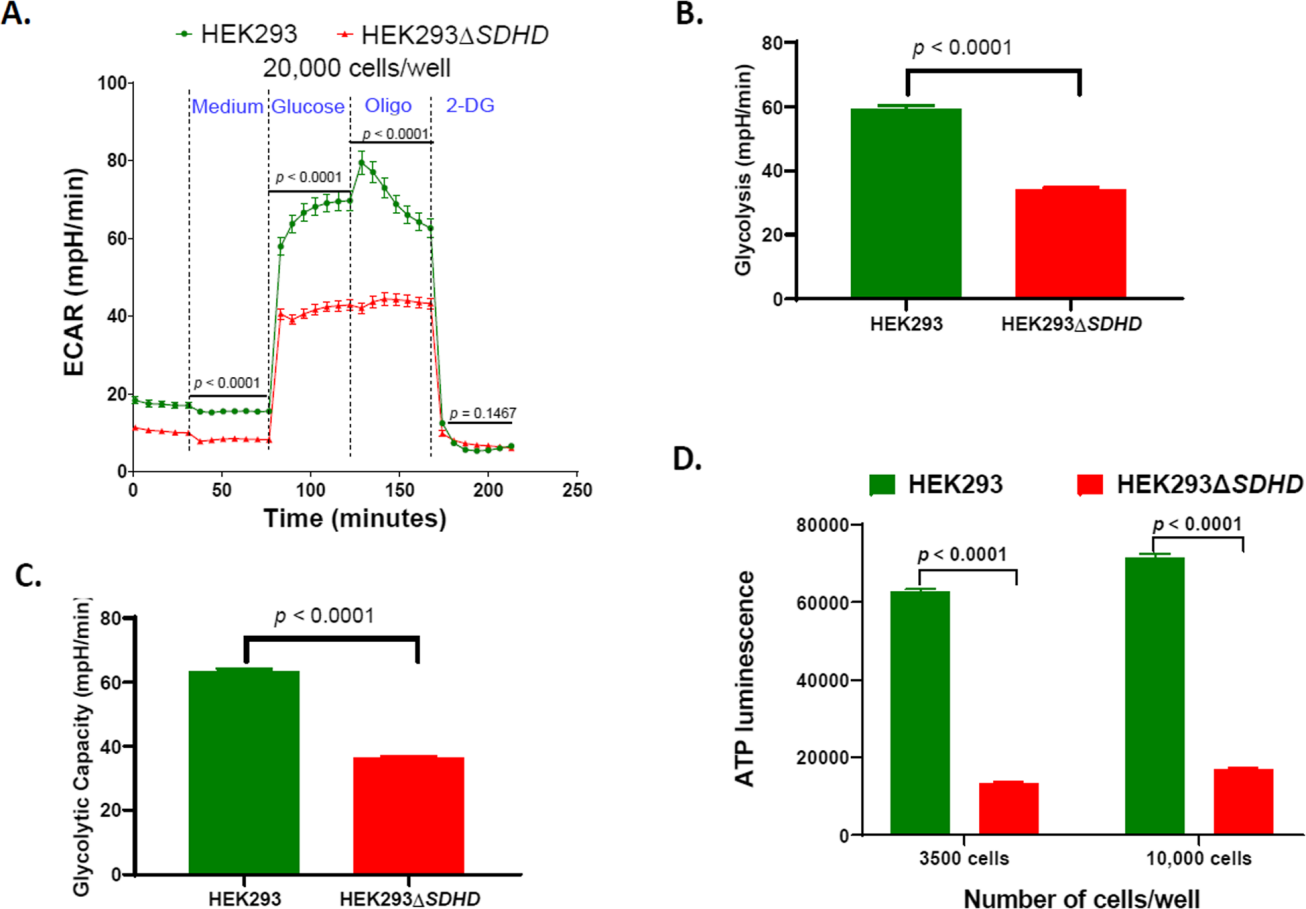
Glycolysis and ATP synthesis of cell lines. ECAR measured using Agilent Seahorse XFe96 analyzer of 20,000 live cells/well (**A**), and calculated glycolysis (**B**) and glycolytic capacity (**C**) are shown. The mean values were compared between the parent HEK293 and the mutant HEK293Δ*SDHD*. ATP synthesis of parent and mutant cells measured using Mitochondrial ToxGlo Assay (**D**) is shown. The measurements are expressed as total luminescence per 3500 or 10,000 cells. Error bars represent the SE of the mean

Extracellular Acidification Rate (ECAR) of intact cells was determined using an Agilent Seahorse XFe96 Analyzer (Fig. 5a). Both glycolysis (*p* < 0.0001; Fig. 5b) and glycolytic capacity (*p* < 0.0001; Fig. 5c) decreased significantly in mutant cells. The findings indicate an association between SDHD function and glycolysis. The mutant cells produced significantly less ATP than parent cells at two different cell densities (*p* < 0.0001 when 3500 or 10,000 cells/well were used; Fig. 5d). Impaired ATP synthesis was consistent with suppressed mitochondrial and glycolytic metabolism.

### Improved respiration and growth of HEK293Δ*SDHD* mutant cells treated with mitochondrial therapeutic idebenone

**Fig. 6:**
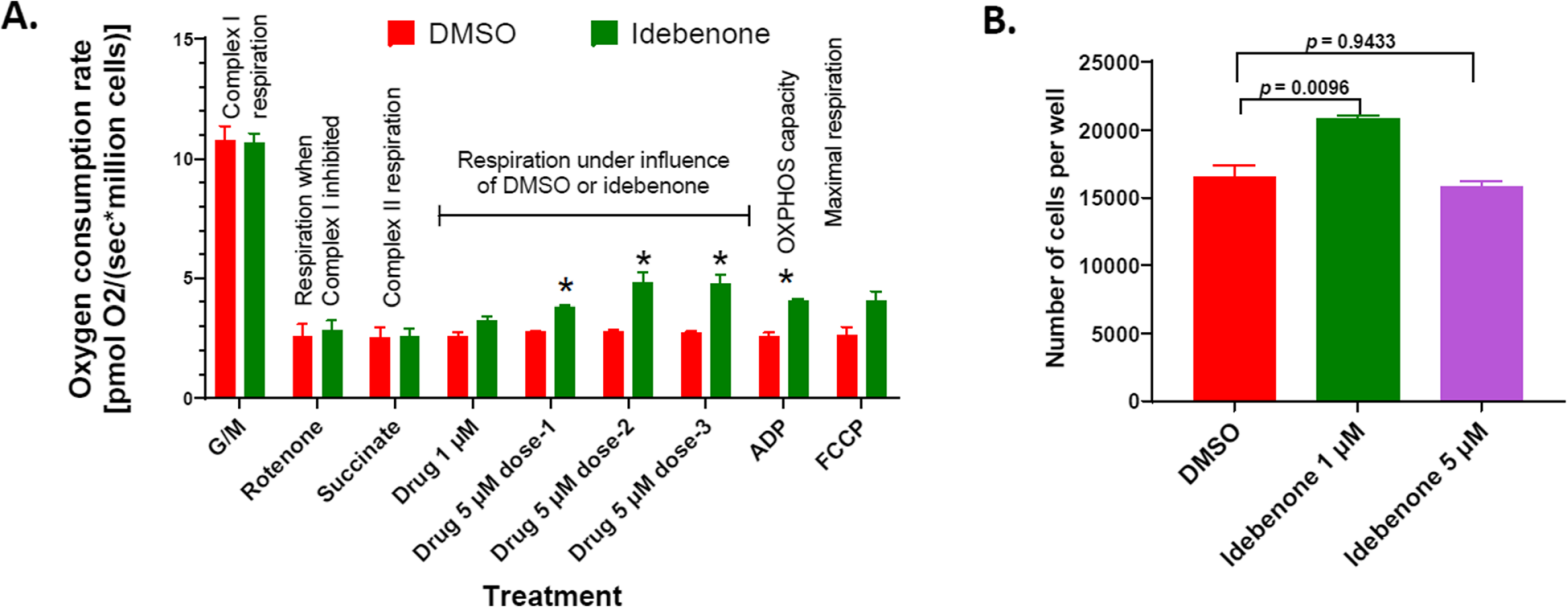
Restoring the defects of the mutant with use of the mitochondrial therapeutic idebenone. The oxygen consumption rates of permeabilized cells treated with DMSO or idebenone are shown (**A**). The rates per million cells in response to injection of substrates, inhibitor, or drug are depicted. The mean oxygen consumption values were compared between the idebenone treatment and DMSO control. The *p* values for the differences between the mean values of DMSO control versus idebenone treatment were 0.8689, 0.7605, 0.9394, 0.1250, 0.0119, 0.0508, 0.0323, 0.0148, and 0.1084 respectively for G/M, rotenone, succinate, 1 μM idebenone, 1^st^ dose of 5 μM idebenone, 2^nd^ dose of 5 μM idebenone, 3^rd^ dose of 5 μM idebenone, ADP, and FCCP. The number of cells recovered from the mutant cultures treated with 1 μM idebenone or DMSO are shown (**B**). Error bars represent the SE of the mean.

Idebenone is a short-chain benzoquinone with greater hydrophilicity [10], which has shown promise as a therapeutic for mitochondrial dysfunction [10–12]. We tested whether idebenone modified oxygen consumption of mutant HEK293Δ*SDHD* cells by Seahorse XFe96 and O2k respirometry. In Seahorse assay, treatment of permeabilized mutant cells with 1 μM idebenone significantly increased basal respiration (*p* < 0.0001; Fig. 4c) and maximal respiratory capacity (*p* < 0.0001; Fig. 4e), but not reserved respiratory capacity (*p* = 0.8847; Fig. 4d). In O2k respirometry assay, in the presence of succinate, idebenone was incrementally added 10 min apart, with concentrations of 1 μM, following by three subsequent injections of 5 μM each (Fig. 6a). Compared to vehicle (DMSO), 1 μM idebenone elicited a non-statistically significant increase in oxygen consumption (*p* = 0.1250; Fig. 6a). Subsequent dose of 5 μM idebenone significantly increased oxygen consumption compared to vehicle in mutant cells (*p* = 0.0119), which peaked after the second dose of 5 μM idebenone (*p* = 0.0508; Fig. 6a). After the final injection of 5 μM idebenone, OXPHOS capacity was measured by adding ADP. Idebenone treated mutant cells had significantly improved OXPHOS capacity compared to the DMSO treated cells (*p* = 0.0148) (Fig. 6a).

We next investigated whether long-term idebenone treatment would influence cell growth. Due to the slow growth of HEK293Δ*SDHD* cells (Fig. 6b), cells were grown for 10 days in growth media where either 1, 5, or 10 μM idebenone was added to the media. After 10 days, 25.4% more cells were recovered from cells treated with 1 μM idebenone compared to the DMSO control (*p* = 0.00959; Fig. 6b). However, treatment with 5 μM idebenone did not improve growth of the mutant (*p* = 0.9433; Fig. 6b), whereas treatment with 10 μM idebenone caused cell death (not shown).

## Discussion

Herein, we successfully used a CRISPR/Cas9 approach to mutate *SDHD*, a Complex II subunit in the inner-membrane region [1, 2], in HEK293 cells, generating the first such model to study the role of this essential Complex II subunit. The INDEL recombination events at the 118-bp site disrupted or altered the whole amino acid sequence downstream of the PAM site. And, mutagenesis procedure did not create any detectable off-target genetic alterations. In agreement with the observed alteration of the *SDHD* DNA sequence, amino acid level expression of the protein SDHD was totally disrupted in the mutant. Moreover, as a result of the *SDHD* mutation, expression of another complex II subunit (SDHB) decreased significantly suggesting interdependency in expression among different subunits of this complex. Quite interestingly, the expression of a complex I subunit (NDUFB8), a complex IV subunit (MT-CO1), and a complex V subunit (ATP5A) also decreased significantly in the mutant suggesting an association in protein expression between complex II and other complexes. Nevertheless, the SDHD mutation did not alter expression of the complex III subunit UQCRC2 suggesting independency of this complex from complex II in terms of protein expression.

Due to the role of Complex II in the ETC and Krebs cycle [1–3] as well as the association of SDHD mutations with disease pathologies [7], understanding the contribution of SDHD to cellular metabolism is warranted. Mutation in *SDHD* disrupted respiration all along the ETC, suppressed glycolysis and overall ATP synthesis, as well as limited cellular growth. Importantly, acute treatment with the synthetic ubiquinone analog idebenone was sufficient to improve Complex II-mediated mitochondrial respiration, OXPHOS capacity, and cell proliferation.

Predictably, mutation of SDHD inhibited Complex II-mediated respiration. Interestingly, however, mutation in SDHD significantly reduced Complex I-mediated respiration as well. Since Complex II drives Krebs cycle in a clock-wise direction by converting succinate to fumarate, we hypothesize that mutation of SDHD slowed and/or disrupted Krebs cycle production of NADH, the substrate for Complex I. Mutation in SDHD also impaired OXPHOS capacity, maximal respiration, and ATP synthesis. Taken together, impairment in the SDHD subunit of Complex II is sufficient to impair overall ETC energy production.

Glycolysis, as determined by ECAR, was significantly reduced in HEK293Δ*SDHD* compared to parent cells. Reduced glycolysis may reflect a lower demand for pyruvate due to disrupted Krebs cycle as a result of mutation in SDHD, representing a potential negative feedback on glycolysis. Also, inhibition of mitochondrial respiration with oligomycin did not elicit a change in glycolysis in mutant cells, further reflecting impaired mitochondrial respiration. In addition to the suppressed metabolism, we also noted significantly slower growth in mutant cells compared to parent cells in culture media, which is likely reflective of the metabolic impairment in mutant cells.

Mutation of SDHD increased apoptosis and necrosis. The impaired glycolysis and mitochondrial respiration may have weakened the cell and cell membrane of the mutant making the cells more apoptotic and susceptible to necrosis. Quite interestingly, the long-term ROS generation declined as a result of SDHD mutation. In general, electrons that do not follow the normal order of the ETC pathway and instead leaked out are eventually transferred directly to O_2_ to generate ROS [13, 14]. In our SDHD mutant, it is possible that impairment of clock-wise direction of Krebs cycle may have decreased the synthesis of NADH and FADH_2_, the two electron donors to the ETC. Thus, limited donation of electrons may have led decreased electron transport through the ETC as well as decreased electron leakage out of the ETC giving rise to decreased ROS generation.

Finally, we explored whether SDHD mutation could be bypassed to improve some of the phenotypes observed. Idebenone, is a short-chain benzoquinone that is more hydrophilic than ubiquinone [10], and has potential as a therapeutic for conditions associated with oxidative stress and mitochondrial dysfunction [10–12, 15–22]. Treatment with varying concentrations of idebenone improved Complex II-mediated oxygen consumption and OXPHOS capacity, suggesting that the ubiquinone analog idebenone is able to substitute for Complex II as an electron donor to improve ETC function. Interestingly, long-term treatment with 1 μM of idebenone increased cell proliferation in HEK293Δ*SDHD* cells whereas 5 μM did not. Idebenone is known to have cell-type specific effects on cell proliferation [23–25]. While an increase in cell proliferation in our mutant HEK293Δ*SDHD* cells with idebenone is likely a result of improved substrate utilization, cell type should be taken into consideration for mitochondrial targeted therapies as to how other process may be influenced.

## Conclusions

We generated a novel mutant of the Complex II subunit SDHD via CRISPR/Cas9 that resulted in severe augmentation to mitochondrial respiration and cell metabolism as well as suppressed growth. This novel tool could be valuable for testing potential mitochondrial-focused therapeutics as well as elucidating other mechanisms regulated by Complex II function.

## Methods

### Cell lines and culture conditions

Human embryonic kidney cell line 293 (HEK293) was kindly provided by Dr. Joseph Ruiz at Enzerna Biosciences (Raleigh, NC). Media and reagents for growing and maintaining cells were purchased from Life Technologies Corporation (Carlsbad, CA). The cells were maintained in Dulbecco’s Modified Eagle’s medium (DMEM) supplemented with 10% (by volume) fetal bovine serum and 1% penicillin-streptomycin. Cells were sustained in a humidified incubator at 37 °C and 5% CO_2_. A 0.25% trypsin-EDTA solution was used for detachment of cells. One Shot Stbl3 Chemically Competent cells of *Escherichia coli* (Life Technologies Corporation) were used for constructing the mutagenesis plasmid. Bacteria carrying the plasmids were maintained in Luria Bertani (Sigma-Aldrich, St. Louis, MO) agar or broth, and sustained in a humidified incubator at 37 °C.

### Construction and validation of *SDHD* mutant

Top and bottom sgRNA sequences for SDHD site were designed and purchased from Integrated DNA Technologies (Skokie, IL). sgRNA oligonucleotides were cloned into plasmid px458 (Addgene, Watertown, MA) and HEK293 cells were transfected with recombinant px458 plasmid using transfection reagent Xfect (Takara Bio USA Inc., Mountain View, CA). Clones with mutation in *SDHD* were picked using procedures described elsewhere [26]. Disruption of SDHD synthesis was validated by Western Immuno-blotting using rabbit polyclonal antibody SDHD (1:1000 in blocking buffer) (Cat# LS-C345301–100; LifeSpan Biosciences Inc., Seattle, WA) and rabbit polyclonal antibodies to β-Actin (1:10,000) (Cat# ab8227, Abcam Inc., Cambridge, MA). A clone missing the protein bands representing SDHD was chosen for further work and designated as HEK293Δ*SDHD* (Fig. 1).

The *SDHD* DNA region including the targeted mutation site was PCR amplified using the primers shown in Table 1 and sequenced by Sanger Sequencing using the same primers. Moreover, the DNA regions encompassing the top three off-targets were PCR amplified and sequenced using the primers shown in Table 1.

Expression of protein subunits representative to each of the five ETC complexes was examined by Western immunoblotting using Total OXPHOS Human WB Antibody Cocktail (Cat# ab110411, Abcam). Since the expression of complexes I and IV subunits was not clearly visible in Western blots with this antibody, the expression of these two complexes was examined separately by Western immunoblotting using antibodies to NDUFB8 (complex I; Cat# ab192878, Abcam) and MT-CO1 (complex IV; Cat# ab203912, Abcam).

### Cell growth and metabolism

Aliquots of 100,000 cells of the parent HEK293 and the mutant HEK293Δ*SDHD* were introduced into 75 cm^2^ flasks each carrying 25 ml growth media. After 4 days of incubation, cell numbers were quantified in triplicates. Generation of reactive oxygen species (ROS) was measured using ROS-Glo H_2_O_2_ Assay (Cat# G8820; Promega Corporation, Madison, WI). Apoptosis and necrosis were assessed using RealTime-Glo Annexin V Apoptosis and Necrosis Assay (Cat# JA1011; Promega). Extracellular Acidification Rate (ECAR) was determined with an Agilent Seahorse XFe96 analyzer [27, 28], and glycolysis and glycolytic capacity were calculated as described elsewhere [28]. Oxygen Consumption Rate (OCR) of saponin-permeabilized cells was assayed with an Agilent Seahorse XFe96 analyzer, and basal respiration, reserve respiratory capacity and maximal respiratory capacity were calculated as described elsewhere [28]. Oxygen consumption of saponin-permeabilized cells [29] was also measured by Oroboros O2k respirometry [30, 31]. Cellular ATP pool was measured using Mitochondrial ToxGlo Assay (Promega Corporation, Madison, WI).

### Restoring the impaired growth and respiration of the mutant

Effectiveness of the potential mitochondrial therapeutic idebenone in restoring the growth defects of the mutant HEK293Δ*SDHD* was evaluated. In this procedure, mutant cells were cultured on 6-well plates at 2000 cells/well in DMEM medium supplemented 1, 5, or 10 μM of idebenone. Fresh media containing the drug was added on days 3, 6 and 9, and the cell counts in wells of triplicates was determined following 10 days incubation. Efficacy of idebenone in improving respiration of permeabilized mutant cells was measured by O2k respirometry as described above with the following modification: subsequent to succinate injection, idebenone was injected into chambers at 1 or 5 μM final concentration. The control groups received DMSO. Moreover, effectiveness of idebenone in improving OCR of permeabilized cells was assessed with the Agilent Seahorse XFe96 analyzer as outlined above with the following modification: cells grown overnight in XFe96 culture plates were washed, and glucose-free DMEM (pH 7.4) supplemented with 10 mM glucose, 10 mM sodium pyruvate, 2 mM glutamine, 20 μg/ml saponin and 1 μM of idebenone was added to the wells prior to incubating for 40 min at 37 °C in a CO_2_-free station. The control wells received the same medium added with DMSO but without idebenone.

### Statistical analyses

Student’s t-tests were performed using Microsoft Excel program (Microsoft, Redmond, WA) or GraphPad Prism 7 (GraphPad Software, San Diego, CA) to compare the means of the parent versus mutant or vehicle control versus idebenone treatment. The ECAR data were analyzed by repeated measures ANOVA using GraphPad Prism 8. Mean differences between groups were considered statistically significant at *p* < 0.05.

## Data Availability

All data generated or analyzed during this study are included in this published article. Any additional data generated during the current study are available from the corresponding author on reasonable request.

## Abbreviations

ETC: Electron transport chain
SDHD: Succinate dehydrogenase subunit D
OXPHOS: Oxidative phosphorylation
ATP: Adenosine triphosphate
CRISPR/Cas9: Clustered regularly interspaced short palindromic repeats/CRISPR-associated protein 9
DMSO: dimethyl sulfoxide
ROS: Reactive oxygen species
OCR: Oxygen consumption rate
